# Coress feedback

**DOI:** 10.1308/003588412X13373405386772

**Published:** 2012-10

**Authors:** Frank CT Smith

**Affiliations:** Programme Director, on behalf of the CORESS Advisory Board

## Abstract

This issue highlights the importance of anatomical orientation, which can sometimes be difficult during bowel anastomosis and stoma formation. The need for good medical communication and an adequate handover, particularly at night and at weekends for patients with medical co-morbidities or following complex surgery, is emphasised in another case.

We are grateful to the clinicians who have provided the material for these reports. The online reporting form is on our website, www.coress.org.uk, which also includes all previous feedback reports. Published contributions will be acknowledged by a ‘Certificate of Contribution’, which may be included in the contributor’s record of continuing professional development.

## Mistaken anatomy 1 (Ref 115)

A 69-year-old male underwent resection of an advanced squamous cell carcinoma of the right mandibular alveolus. Temporary tracheostomy, selective neck dissection, segmental mandibulectomy, dental extractions, reconstruction with a right fibula-free flap and insertion of an open gastrostomy tube were planned.

Timings for the procedure proved difficult. The first provisional date for surgery was declined by the patient. The operation date was therefore brought forward by one week and preceded by pre-operative assessment. There were no beds available on the day prior to surgery so the patient was advised to attend at 09.30 on the morning of the procedure. An intensive therapy unit bed had to be secured. The planned start time of the operation was 10.45 but knife to skin occurred at 12.30. The procedure was complicated and took until 19.00. Due to staff shortages and lack of availability of gastrointestinal surgeons, the planned gastrostomy was deferred and a nasogastric (NG) tube placed in situ. Post-operative x-rays demonstrated that the NG tube was incorrectly positioned and three further (futile) attempts were made to re-site this.

The following morning, the patient was placed on the emergency list for gastrostomy, which was postponed due to other cases, until the early evening when he was taken back to theatre and a gastrostomy tube inserted by the on-call surgical registrar, in a lengthy open procedure. Early feeding via the gastrostomy was commenced but the patient failed to improve, developing a pyrexia and gradually increasing C-reactive protein over the next 3 days. When he developed diarrhoea and epigastric pain on the fourth post-operative day, an abdominal computed tomography (CT) scan was undertaken, with contrast introduced down the gastrostomy. This revealed the gastrostomy to be sited in the mid-transverse colon with some extravasation of contrast. At further laparotomy the feeding tube was removed from the colon and because of some local contamination, a tranverse loop colostomy was undertaken and the gastrostomy re-sited appropriately.

Subsequently the patient made an uneventful recovery, in-hospital stay prolonged while he learned to manage his stoma, which was successfully reversed three months later.

### Reporter’s comments

These problems started with late admission of the patient on the morning of major procedure and were compounded by a string of other problems: no ITU bed was available initially; there was a late start with a late finish and no availability of other surgeons. The gastrostomy was therefore subsequently undertaken inappropriately as an elective procedure on an emergency list and delegated to the on-call trainee who didn’t request help when he ran into difficulties.

### CORESS comments

Complex cases require meticulous pre-operative planning. Day-of-surgery admissions are feasible and save hospital resources, provided protocols are adhered to. In a case such as this, some surgeons may have considered pre-emptive use of a percutaneous gastrostomy. If the trainee experienced problems during the latter procedure help should have been sought at an early stage.

## Mistaken anatomy 2 (Ref 118)

A 73-year-old man presented with a one-year history of a change in bowel habit. He underwent a colonoscopy undertaken by a nurse endoscopist where a very large polyp, which could not be negotiated, was found in the rectosigmoid colon. Biopsies confirmed a tubulovillous adenoma (TVA) with high-grade dysplasia. CT scan demonstrated a 6cm mass in the rectosigmoid, but with no sign of invasion or metastatic disease.

The MDT decided that further endoscopy should be undertaken by a consultant to determine a) if the lesion were truly malignant, b) its height and c) if it were removable endoscopically. The lesion was not resectable endoscopically and was intussuscepting so that the height could not be accurately determined. A second set of biopsies again showed high-grade dysplasia in a TVA. The patient was counselled and scheduled for resection. Before operation could be undertaken, however, the patient became unwell with signs and symptoms of large bowel obstruction.

The patient was admitted from clinic and resuscitated. The next day it was decided that he should undergo defunctioning colostomy as an emergency to prevent perforation and should undergo examination under anaesthetic to determine the height of the lesion in case radiotherapy should be required. The patient was marked for a transverse loop colostomy by the stoma nurses to allow full colonic decompression and to avoid the obstructing lesion.

As it was a weekend, I discussed this with the gastrointestinal consultant on call and arranged that this would be done on the emergency list under his care. The operation was carried out by an experienced trainee with an interest in colorectal surgery. The consultant was not present in the operating theatre but was on site and readily available if needed.

The operation appeared proceed without problem. I saw the patient 48 hours after the operation. He was well and had a pink healthy stoma, with a bridge, in the right upper quadrant. He was reviewed daily. 72 hours post-operatively, he developed a cardiac arrhythmia and was transferred to the coronary care unit (CCU). This was thought to be due to magnesium depletion (not uncommon following obstruction and a large TVA). He required magnesium infusion and, at one stage, cardioversion. Shortly afterwards, the stoma developed a high output (2–3 litres/24hr) and skin excoriation. I realised that something was ‘wrong’, though I was not sure what it was and arranged for contrast to be instilled down each limb of the stoma via Foley catheters. The subsequent x-ray suggested that one catheter was in the stomach and the other in the duodenum. This was confirmed by CT, which demonstrated that the distal stomach had been brought out and fashioned into a loop stoma.

I was dismayed and discussed the safest way forward with my consultant colleagues. We undertook urgent laparotomy as a two-consultant procedure, closing the gastric stoma around a Foley catheter (as we were concerned about healing), placed a feeding jejunostomy and undertook a Hartmann’s procedure, as when the intussuscepted sigmoid polyp was reduced this resulted in an intra-intussusception perforation. I had a very difficult conversation with the patient and family explaining what had happened. I made an unreserved apology for the error and promised a full inquiry. Following this the patient made a slow but steady recovery and was eventually discharged home. Final histology showed no evidence of invasion. A hospital serious untoward incident inquiry was held.

### Reporter’s comments

The patient’s delayed presentation and diagnostic difficulties led to an urgent procedure. During surgery the anatomy was misidentified by the trainee who failed to identify greater and lesser omentum and taeniae coli and didn’t request assistance.

### CORESS comments

This case reinforces the message that a call for help (or even just a quick check: ‘Is this ok?’ ‘Does this look right’) is not an admission of failure but good professional practice. Trainees may not always understand all the steps in a procedure despite having been taken through it several times. If a patient does not progress as one might expect after an operation, question what happened during the procedure. Good communication with immediate explanation and apology for the error helped to resolve potential conflict. Where corrective surgery has to be undertaken to resolve a problem, a two-consultant procedure is good clinical practice.

## Inadequate handover and weekend cover (Ref 125)

Our NHS trust, a medium-sized one, is based across two hospital sites approximately 12 miles apart. One of these sites has an elective operating facility, used mostly by orthopaedics, located away from the main hospital. This is a self-contained unit with capacity to deal with all stages of the patient’s journey, from pre-admission to post-operative rehabilitation. The unit is fully staffed and functional during the working week but is run just by ward nurses during the weekend.

A 72-year-old lady with medical co-morbidities underwent a total knee replacement on a Friday. Soon after surgery it was noted that she had excessive bleeding from the wound, requiring blood transfusion. There was no routine weekend ward round by the on-call orthopaedic team and at no point was an orthopaedic surgeon called to attend the patient. The surgeon who performed the operation, a locum consultant with some experience and seniority, was also not contacted.

The patient was eventually attended by a cardiologist because she had chest pains and it was then noted that she had sustained a myocardial infarction. She was transferred to the CCU, where she spent several days. She was eventually discharged from hospital. On follow-up with orthopaedics, it was noted that she had a stiff knee due to the lack of physiotherapy input during the time she was away from an orthopaedic ward. This had to be addressed with a manipulation under anaesthetic. A few weeks later she presented to another NHS trust with chest pain and died on arrival in accident and emergency.

### Reporter’s comments

This significant operative procedure was performed on a Friday afternoon by a locum consultant who had no nominated junior staff to cover his patients. No weekend ward rounds were undertaken at the elective facility and there was lack of on-call surgical input because of communication breakdown. No handover was undertaken and neither the locum consultant nor any of the on-call team made enquiries about the wellbeing of the patients at the elective centre. Issues were raised concerning nursing communication, especially with the on-call doctors.

### CORESS comments

The operating surgeon or consultant in charge of the patient has a professional duty to ensure continued and sustained care for his or her patients and any inpatient undergoing surgery should be reviewed on the following day. This is facilitated by modern teamworking practices. Adequate medical handovers should be conducted when there are shift changes and particularly at night and at weekends.

If there is downgrading of medical or surgical cover at weekends, then perhaps scheduling of major surgical cases on a Friday may be inappropriate.

## Forgotten tourniquet (Ref 132)

A 64-year-old man sustained injuries to the pulps of his non-dominant middle and ring fingers on a hedge trimmer. He was taken to theatre and his fingers were washed, debrided and sutured under local anaesthetic ring blocks. During the procedure, the fingers of surgical gloves were rolled down to act as ring tourniquets on each finger, to provide a bloodless operative field. An artery clip was used to secure the tourniquet at the base of the middle finger and this was removed on completion of surgery, after which dressings were applied to the hand. The patient was subsequently discharged with the hand dressed, with simple analgesia and oral antibiotics.

In the ensuing post-operative period he suffered significant discomfort. So much so, that he presented to his GP and local NHS walk-in centre for review on three occasions. Unfortunately, his dressings were not taken down on any of these occasions and he was sent home with stronger analgesia each time. He finally he had his dressings taken down on review in the dressing clinic when the tourniquet was discovered, still in-situ on his ring finger. This was removed. Remarkably, the ring finger was congested but viable, although a reduction in sensation distal to the site of the tourniquet was noted. On further review five days later, the congestion in the finger had resolved but sensory loss persisted.

### Reporter’s comments

This case occurred because there was non-adherence to trust policy of not using glove fingers as ring tourniquets. Only one artery clip was used on one of the ring tourniquets. This ensured that this tourniquet was taken off at the end of the operation to facilitate dressing, at which time the other tourniquet was missed. The use of tourniquets was not recorded on the theatre whiteboard, failing to prompt removal on completion of the procedure. Failure to take down the patient’s dressings to examine the hand for a source of persisting pain, by both GP and NHS walk-in centre staff, compounded the error.

### CORESS comments

Glove fingers should not be used as ring tourniquets under any circumstances. Instead, brightly coloured tourniquets that are easily apparent should be employed. The whole theatre team should have been involved to ensure that the tourniquet was not forgotten at the end of the procedure and the outcome should have been avoided if World Health Organization checks had been used. Use of a tourniquet should have been recorded on the theatre whiteboard. Day-case patients should have recourse to an emergency contact number on discharge. There is an onus on the reviewing clinician and triage nursing staff to undertake a full and appropriate examination to determine a source of pain.

